# Cytotoxic Effects and Anti-Angiogenesis Potential of Pistachio (*Pistacia vera* L.) Hulls against MCF-7 Human Breast Cancer Cells

**DOI:** 10.3390/molecules23010110

**Published:** 2018-01-05

**Authors:** Maryam Seifaddinipour, Reyhaneh Farghadani, Farideh Namvar, Jamaludin Mohamad, Habsah Abdul Kadir

**Affiliations:** 1 Institute of Biological Sciences, Faculty of Science, University of Malaya, Kuala Lumpur 50603, Malaysia; m.seyfadini@gmail.com (M.S.); jamal@um.edu.my (J.M.); 2 Department of Molecular Medicine, Faculty of Medicine, University of Malaya, Kuala Lumpur 50603, Malaysia; r_farghadani@yahoo.com; 3 Faculty of Medicine, Mashhad Branch, Islamic Azad University, Mashhad 917568, Iran

**Keywords:** apoptosis, *Pistachio vera*, ethyl acetate extract, anticancer activity, cytotoxicity, angiogenesis, breast cancer

## Abstract

Pistachio (*Pistacia vera* L.) hulls (PVLH) represents a significant by-product of industrial pistachio processing that contains high amounta of phenolic and flavonoid compounds known to act as antioxidants. The current study was designed to evaluate the anti-tumor and anti-angiogenic potentials of PVLH extracts. The cytotoxic effects of hexane, ethyl acetate, methanol, and water PVLH extracts toward human colon cancer (HT-29 and HCT-116), breast adenocarcinoma (MCF-7), lung adenocarcinoma (H23), liver hepatocellular carcinoma (HepG2), cervical cancer (Ca Ski), and normal fibroblast (BJ-5ta) cells were assessed using a MTT cell viability assay. Apoptosis induction was evaluated through the different nuclear staining assays and confirmed by flow cytometry analysis. Anti-angiogenic activities were also determined using chorioallantoic membrane (CAM) assay. PVLH ethyl acetate extracts (PVLH-EAE) demonstrated a suppressive effect with an IC_50_ value of 21.20 ± 1.35, 23.00 ± 1.2 and 25.15 ± 1.85 µg/mL against MCF-7, HT-29 and HCT-116, respectively, after 72 h of treatment. Morphological assessment and flow cytometry analysis showed the potential of PVLH-EAE to induce apoptosis. PVLH-EAE at the highest concentration demonstrated significant inhibition of angiogenesis as comparing with control group. Also the expression of Bax increased and the expression of Bcl-2 decreased in treated MCF-7 cells. Thus, the apoptosis induction and angiogenesis potential of PVLH-EAE make it to be the most suitable for further cancer research study to deal with selective antitumor active substances to human cancers especially breast cancer.

## 1. Introduction

Pistachio (*Pistacia vera* L.) is native of Central and West Asia and distributed throughout the Mediterranean basin [1]. Pistachio nuts have recently been ranked among the first 50 highest antioxidant food products and a rich source of phenolic compounds [2,3]. Pistachio nuts are often used after removing the hull, which thus represents a significant by-product of industrial pistachio processing [4]. Previous phytochemical investigations have revealed that the total content of phenolic compounds in pistachios is significantly higher in hulls than in seeds [5].

Cancer is caused by environmental factors such as carcinogens, viruses, chemicals, and radiation as well as by a genetic history such as cell line mutations leading to malignant growth, invasion, and metastasis [6]. Programmed cell death deficiency is a key driver of both cancer progression and drugs responsiveness [7]. Apoptotic pathways are being regulated by a number of gene families [8,9] through intrinsic or extrinsic pathways [10]. The intrinsic pathway relies on the release of cytochrome c from mitochondria to form an apoptosome, which interacts with apoptotic protease activating factor 1 and procaspase-9 through cleavage to give an active form of caspase-9 [11]. Apoptosis is also under the control of various proteins/genes which are categorized into two main groups, namely pro-apoptotic proteins/genes that have positive effect on apoptosis to make the cellular process go further and anti-apoptotic having a negative effect and blocking apoptosis [4]. Although chemotherapy-induced apoptosis is the main approach of numerous anti-cancer therapies, many drugs have been implicated in the emergence of treatment resistance and side effects [12]. The discovery of potent drugs targeting apoptosis signal transduction is warranted to ameliorate clinical consequences in cancer therapy [12].

There are a few studies about pistachio’s anticancer effects. The hull of this plant is used in traditional medicine as stomach pain healer, prevent of diarrhea, and improve hemorrhoids [13]. Different parts of pistachio possess in vitro radical scavenging properties [14] that protect LDL from being oxidized and will thus have direct beneficial effects on atherosclerosis and overall heart disease risk [15,16,17]. He et al. reported that mastic gum, a resinous exudation obtained from the stem and leaves of *Pistacia lentiscus* trees, was a “conglomeration of effective anticancer drugs” to support the anticancer activities of mastic gum and its major constituents and highlighting the various molecular mechanisms through which the triterpenoids work their anti-cancer magic [18]. Moreover, pistachio hulls have been shown to exhibit antioxidant, antimicrobial, enzyme inhibitory and radical scavenging effects [16]. Pistachio skins contain epicatechin, quercetin, naringenin, luteolin, kaempferol, cyanidin-3-*O*-galactoside and cyanidin-3-*O*-glucoside [17]. Quercetin, the most frequently studied flavonoid, has been shown to have anticancer properties by in vivo and in vitro experiments [3]. Many studies have described the beneficial effects of dietary kaempferol in reducing the risk of chronic diseases, especially cancer [5]. Epidemiological studies have shown an inverse relationship between kaempferol intake and cancer [19]. Epicatechin is also a known novel anticancer drug [20]. The present study was carried out to assess the cytotoxic potential of pistachio hulls (PVLH) against several cancer cell lines, with the particular aim of evaluating the antiangiogenesis and apoptosis induction of the best extract against the most sensitive cancer cell line.

## 2. Results

### 2.1. Cell Viability Effect of PVLH against Cancer Cells

The cytotoxic potential of four different extracts of PVLH (hexane, ethyl acetate, methanol and water) was evaluated on several cancer cell lines by the MTT assay in triplicate. IC_50_ values for the extracts used in this study were shown in Table 1. The ethyl acetate extract (PVLH-EAE) displayed a significant inhibitory effect towards both breast and colon cancer cells after 72 h. The IC_50_ of PVLH-EAE against MCF-7, HT-29 and HCT-116 cells were calculated as 21.20 ± 1.35, 23.00 ± 1.2 and 25.15 ± 1.85 µg/mL, respectively.

### 2.2. Cytotoxic and Cell Viability Effect of PVLH-EAE toward MCF-7

**Figure 1:**
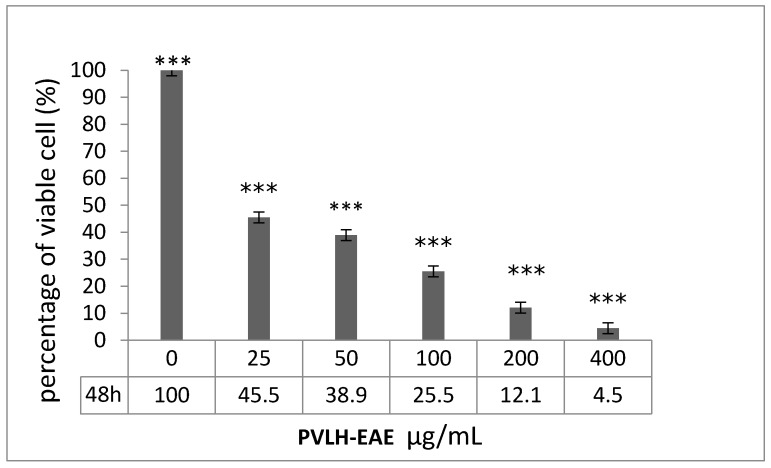
Percentage of viable MCF-7 cells treated with PVLH-EAE after 48 h. Increasing concentrations of PVLH-EAE lead to a lower percentage of viable cells (***: *p* value < 0.001). All in vitro experiments were performed in triplicate and expressed as the mean ± standard deviation.

The toxicity effects of PVLH-EAE on viability of MCF-7 cells were checked by MTT and trypan blue assays. In trypan blue assay, the percent of viable MCF-7 cells were calculated after 48 h incubation with different concentrations of PVLH-EAE. Our results demonstrate a concentration-dependent decrease in cell viability after 48 h exposure to PVLH-EAE (Figure 1). At the lowest concentration of PVLH-EAE (25 µg/mL), less than half of cells survived (45.5%). At concentrations greater than 25 μg/mL, the viability of the cells was less than 40%. At the highest concentration of PVLH-EAE, more than 95% of the cells were dead and the survival rate of MCF-7 cells was 4.5%.

**Figure 2:**
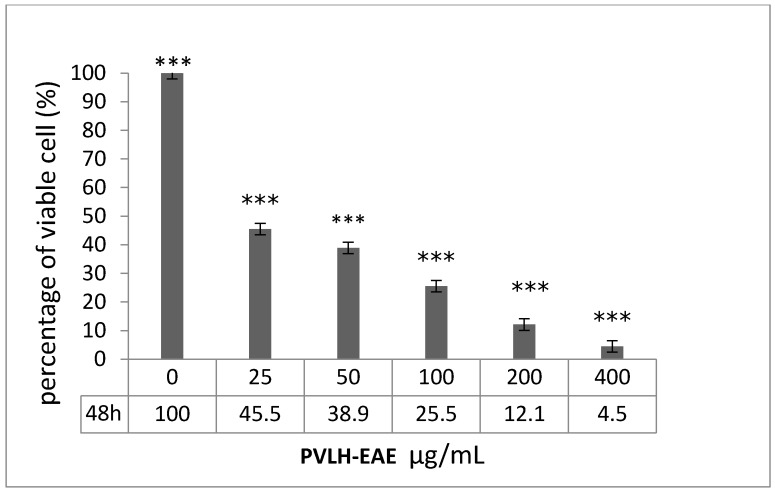

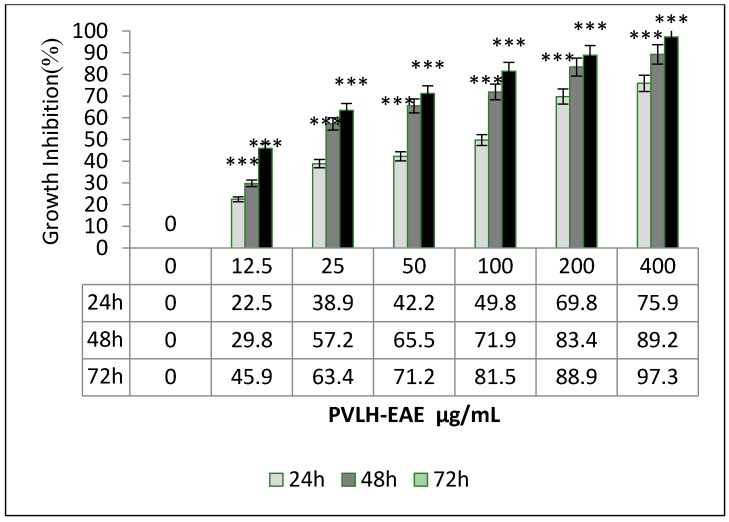
Growth inhibition effects of PVLH-EAE on MCF-7 in different time (24, 48 and 72 h) and concentration (12.5, 25, 50, 100, 200 and 400 μg/mL). The longer incubation time and increasing concentrations of PVLH-EAE lead to more percent inhibition of cell growth (***: *p* value < 0.001). All in vitro experiments were performed in triplicate and expressed ad the mean ± standard deviation.

The MCF-7 cell viability was determined for different concentrations (12.5, 25, 50, 100, 200 and 400 µg/mL) of PVLH-EAE and incubation times (24, 48, and 72 h) by a MTT assay. The cell viability decreased in a time and dose dependent manner. We observed that at the highest concentration of PVLH-EAE (400 μg/mL) after 72 h of exposure, less than 3% of MCF-7 cells were viable (Figure 2). IC_50_ values of 21 μg/mL were calculated at 48 h exposure time to PVLH-EAE for both the trypan blue and MTT assays. Our results showed that PVLH-EAE affected MCF-7 cells viability even at low concentration.

### 2.3. Apoptotic Morphological Changes

**Figure 3:**
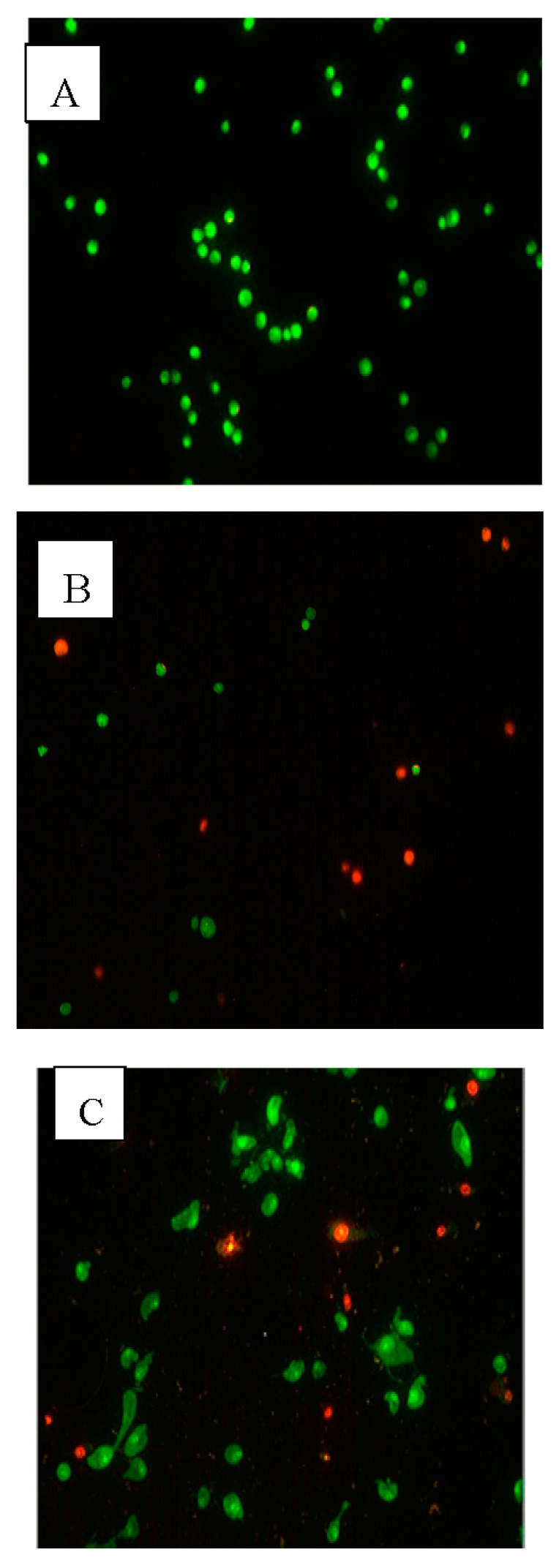
Fluorescent images of MCF-7 cells stained by AO/PI. (**A**) Control cells (×200); (**B**) treated with 0.1% DMSO for 48 h (×200); (**C**) treated with 25 μg/mL PVLH-EAE for 48 h (×200).

**Figure 4:**
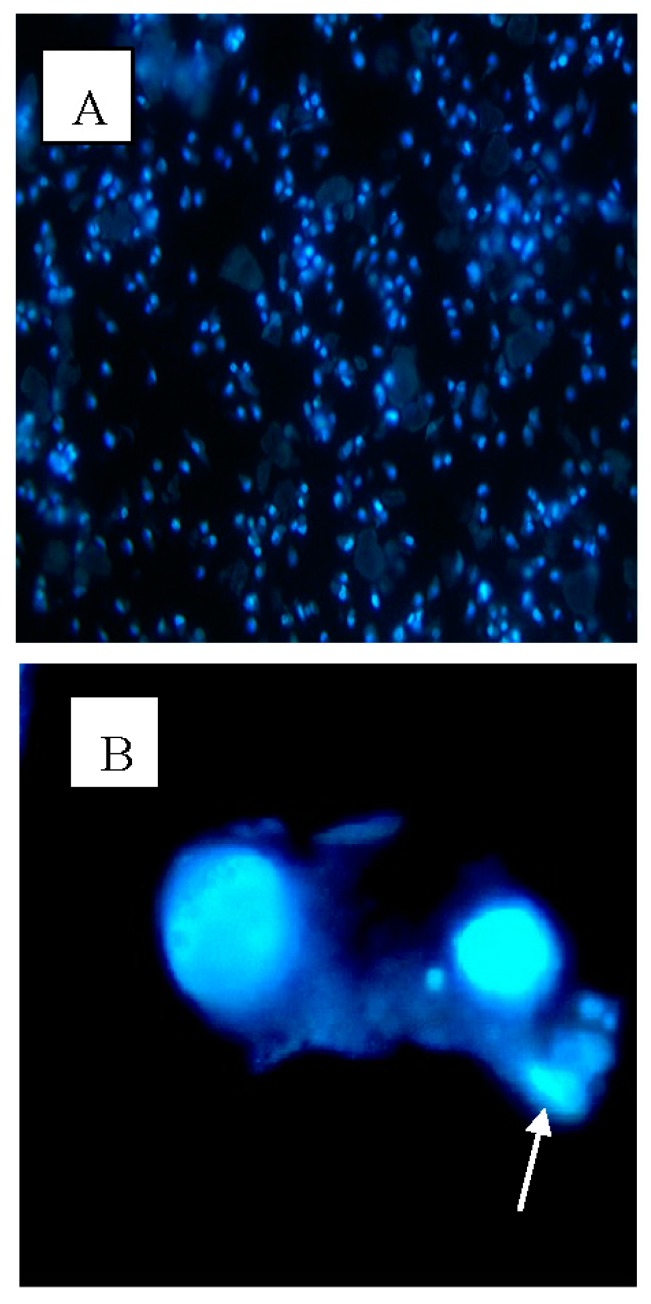
Fluorescent images of MCF-7 cells stained by Hoechst 33,342. (**A**) Control cells (×200); (**B**) treated with 25 μg/mL PVLH-EAE for 48 h (×400) (arrow show bleb formation).

Table 2 present the results obtained from the acridine orange/propidium iodide staining (AO/PI) staining of MCF-7 cells. From this data, we can see that PVLH-EAE has time-dependent effects on cell viability. The results show decreased viability with higher numbers of apoptotic cells at all three time points (24, 48 and 72 h) of treatment with 0.1% DMSO and 25 μg/mL PVLH-EAE. After 24 h treatment with DMSO the viability of cells decreased to 93.7%, while, at the same time the viability of PVLH-EAE treatment decreased to 68.9%. At 72 h, in DMSO treatment, the viability decreased to 87.3%, while, at the same time the viability of PVLH-EAE treatment decreased to 32.1%. In addition, the AO/PI (Figure 3) and Hoechst 33,342 (Figure 4) staining revealed that the PVLH-EAE induces apoptotic morphological changes in treated cells such as bleb formation.

### 2.4. Flow Cytometry Analysis

**Figure 5:**
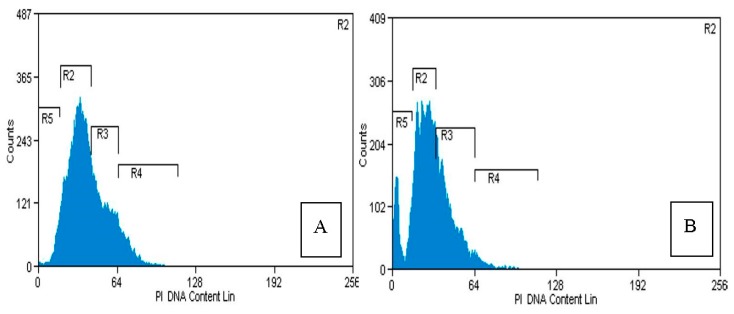
Cell cycle analysis of MCF-7 cells. (**A**) Control cells; (**B**) treated with 25 μg/mL PVLH-EAE for 48 h (R5: Sub G1, R2: G1, R3: S, R4: G2/M).

The apoptotic morphological change described above were also confirmed with a flow cytometric analysis. After the MCF-7 cells were treated with PVLH-EAE at the designated concentration (25 μg/mL) for 48 h, they were harvested and stained with propidium iodide, and the cell populations of each phase were counted by flow cytometry. As shown in Figure 5, the sub-G1 population, which indicated apoptotic cells, increased from 1% at 0 μg/mL (control) to 25.53% at 25 μg/mL, after exposure to PVLH-EAE for 48 h. Although the G1 population decreased along with an increase of sub-G1, the other portions of non-apoptotic cells did not show a significant change.

### 2.5. Real Time PCR Results

**Figure 6:**
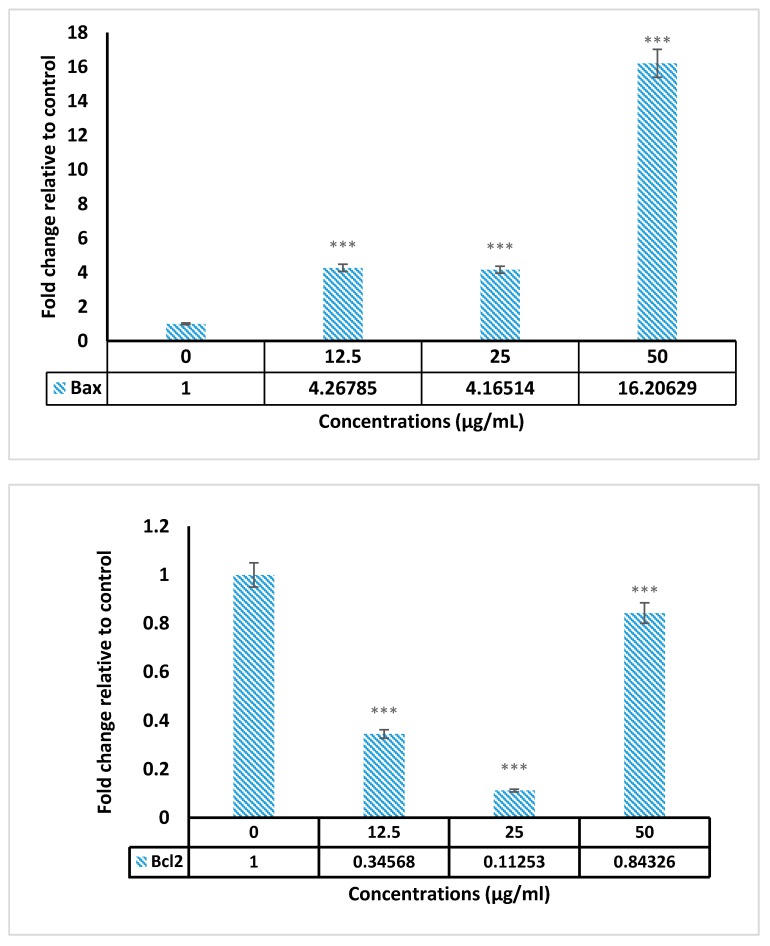
The Bax and Bcl-2 gene expression of MCF-7 cells treated with 12.5, 25 and 50 μg/mL PVLH-EAE. (***: *p* value < 0.001).

The Bax and Bcl-2 genes expression were assessed using real time PCR in treated cells with 12.5–50 µg/mL of PVLH-EAE in 24 h. Results indicated that expression of Bax up regulated in treated cells. But the expression of anti-apoptotic Bcl-2 decreased in dose 12.5 and 25 but slightly increased in dose 50 µg/mL (Figure 6).

### 2.6. Chorioallantoic Membrane (CAM) Assay

**Figure 7:**
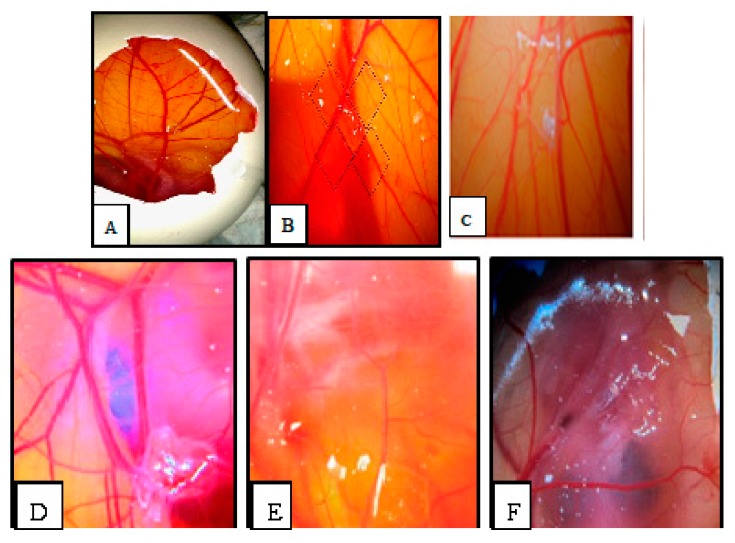
(**A**) Image of CAM with gelatin sponge; and (**B**) Square which counting was done there (right); (**C**) Control, (**D**) 12.5; (**E**) 25 and (**F**) 50 μg/mL concentrations of PVLH-EAE.

**Figure 8:**
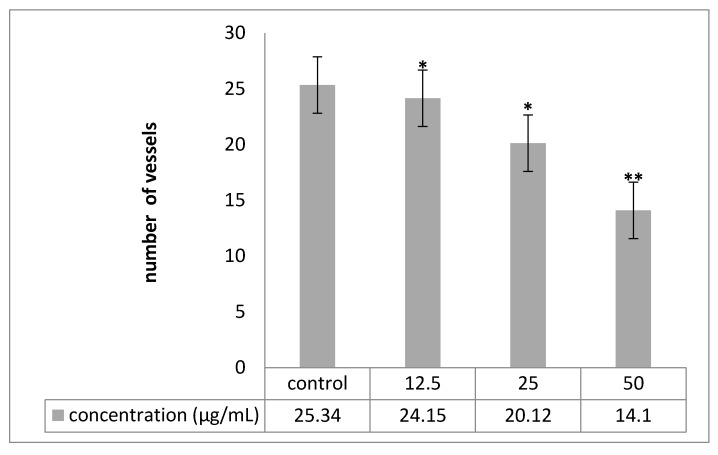
Comparison of number of new vessels in four groups. Treated groups compared with control group (* *p* < 0.05, ** *p* < 0.001). Experiments performed in triplicate.

**Figure 9:**
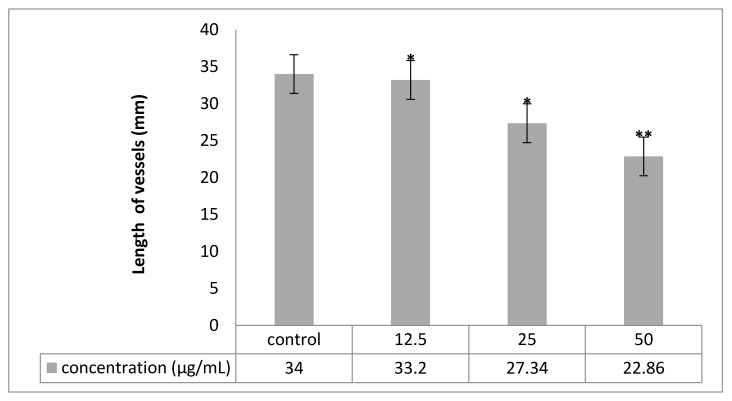
Comparison of length of new vessels in four groups. Treated groups compared with control group (* *p* < 0.05, ** *p* < 0.001).

**Figure 10:**
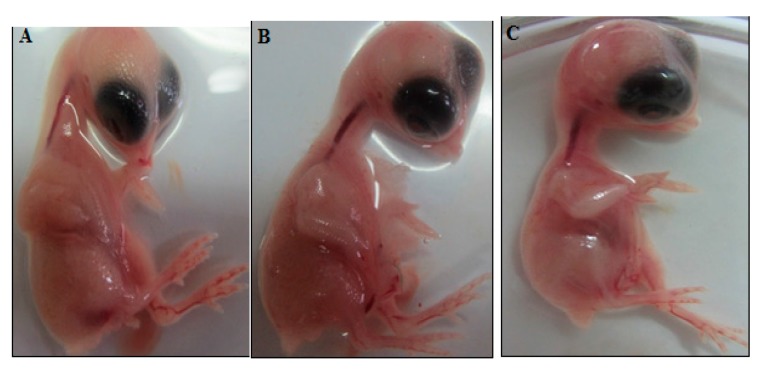
Morphometric changes: No disorder on development of chick embryo was seen. (**A**) Control, (**B**) 25 µg/mL, (**C**) 50 µg/mL.

Angiogenesis was evaluated by CAM assay. The angiogenesis indicators (number and length of the newly formed arterioles) were significantly (*p* < 0.05) decreased with low concentration treatment of PVLH-EAE versus control. The eggs treated with higher concentration of PVLH-EAE (50 μg/mL) showed significantly reduction of vessels formation (*p* < 0.001) (Figure 7, Figure 8, Figure 9 and Figure 10). Based on the results from CAM model, PVLH-EAE at the highest concentration demonstrated significant inhibition of angiogenesis as comparing with control group. No significant changes in chick morphology (both the Crown Rump length and weight) was observed in control group and treated group with 50 μg/mL concentration of PVLH-EAE at the end of study (Figure 10) “(Data not shown)”.

## 3. Discussion

One of the hallmarks of cancer is the ability of malignant cells to evade apoptosis [18]. Thus, a comprehensive perception of apoptotic signaling pathways involved is of crucial importance for discovery of target selective therapeutics. Having examined PVLH as a newly *Pistachio vera* red hull extract, we found this agent to be potentially cytotoxic to colon and breast cancer cells. Importantly, the cytotoxicity to normal cells studied in this research is relatively insignificant, suggesting that PVLH exhibits selective activity toward different cancer cells. This feature can be considered as a prominent property of this extract in cancer cell treatment. Our findings provide strong molecular evidence for potential cytotoxic properties of PVLH to breast cancer cells through various mechanisms. To address whether PVLH induces cytological alterations in the breast cancer cell lines, we also monitored cell cycle in PVLH-treated cells. Antioxidant, anti-microbial and antimutagenicity activities of pistachio (Ahmadaghaei variety) green hull extracts (crude and purified extracts) were studied by Rajaei et al. [16]. They used different solvents for determining of the best solvent for extraction of phenolic compounds from pistachio green hull. Water and acetonitrile with 49.32 and 6.22 (mg of gallic acid equivalents/g sample) were the best and the worst solvent in the extraction of phenolic compounds, respectively. Based on our results PVLH ethyl acetate extracts demonstrated a suppressive effect on breast cancer cells. Their results of antimutagenicity test showed that phenolic compounds of pistachio green hull have antimutagenicity activity against direct mutagen of 2-nitrofluorene [16].

Barreca et al. in their research extracted the hulls of ripe pistachios with two organic solvents (ethanol and methanol) and characterized for phenolic composition, antioxidant power and cytoprotective activity. In RP-HPLC-DAD-FLU separation they identify 20 derivatives, the most abundant being gallic acid, 4-hydroxybenzoic acid, protocatechuic acid, naringin, eriodictyol-7-*O*-glucoside, isorhamnetin-7-*O*-glucoside, quercetin-3-*O*-rutinoside, isorhamnetin-3-*O*-glucoside and catechin. Methanol extraction gave the highest yields for all classes of compounds and presented a higher scavenging activity in all the antioxidant assays performed. The same was found for cytoprotective activity on lymphocytes, lipid peroxidation and protein degradation. Their findings highlight the strong antioxidant and cytoprotective activity of the extract components [21], the methanol extract having a 1.7-fold higher phenolic content than that of ethanol. In our results ethyl acetate extracts have more cytotoxic effect and maybe the anticancer mechanism is more related to antiangiogenesis than antioxidant effects.

Results from cytotoxicity showed that the PVLH-EAE extracts had no toxic effect on the normal cells. On the other hand, they could significantly affect all five human cancer cell lines. The MCF-7 cell line was the most sensitive and HepG2 was the most resistant.

Fathalizadeh et al. showed that pistachio (*Pistacia vera* L.) hull extract treatment reduced cell viability (IC_50_ ~ 0.3 mg/mL) in a dose-dependent manner and flow cytometric analysis revealed that the extract significantly induced apoptosis in HepG2 cells by the regulation of apoptosis-related genes including TNF, BCL2, IAP, TRAF, and caspase families [22]. In our results the IC_50_ for HepG2 cells was more than 100 µg/mL so for mechanistic study we continue with more sensitive cells (MCF7).

In addition, induction of apoptosis is a useful approach in cancer therapy. In apoptotic cells, several cellular and molecular biological features, such as cell shrinkage, DNA fragmentations, and activation of the caspase cascade, are exhibited [23]. Cell membrane blebbing is one of the typical early apoptotic characteristics which were found when treated cells were observed. Cytotoxicity of PVLH-EAE was evaluated by growth inhibition. When the growth inhibited cells were stained with AO/PI and Hoechst 33,342 apoptotic cell death was observed in time depended manner in all cultures. These results suggested that PVLH-EAE caused irreversible cell damages in cultured cells.

Regulation of the cancer cell cycle is one strategy in the development of anticancer drugs. The result of cell cycle analysis determined by flow cytometry analysis also showed that PVLH-EAE can induce apoptosis in MCF-7 cells without cell cycle arrest.

According to Mirian et al. results *P. vera* extracts inhibited VEGF-induced tube-like structures at concentration of 250 μg/mL to 87.59 ± 1.8. The IC_50_ values of the antiangiogenic effects of these extracts were 118.86 ± 5.14 [24]. In this research the eggs treated with higher concentration of PVLH-EAE (50 μg/mL) showed significantly reduction of vessels formation. Based on the results from CAM model, PVLH-EAE at the highest concentration demonstrated significant inhibition of angiogenesis as compared with control group.

## 4. Materials and Methods

### 4.1. Plant Material and Extraction

**Figure 11:**
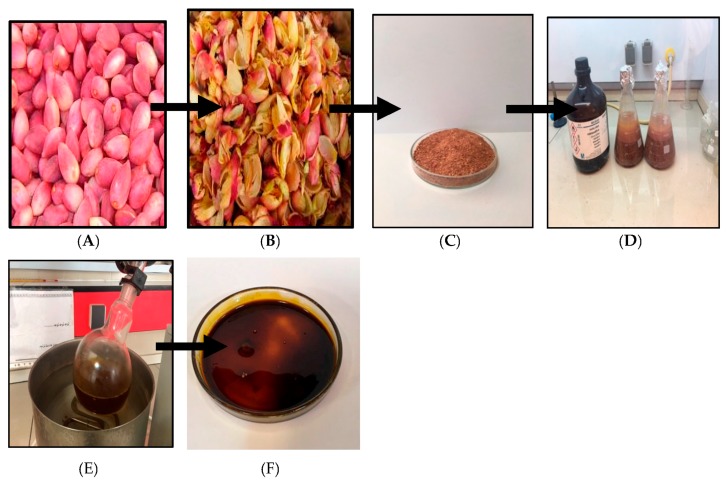
Extraction method of PVLH (**A**) fresh; (**B**) drying; (**C**) powder; (**D**) maceration; (**E**) evaporation and (**F**) extract (PVLH).

The pistachio red hulls (PVLH) were collected from Kerman Province, Iran and identified by Dr. Yong Kien Thai at the Herbarium in the University of Malaya Institute of Biological Science. A voucher specimen was deposited as number KLU48697. Subsequent drying and powdering of hull (5 kg), maceration with hexane, ethyl acetate, methanol, and water (3 × 2500 mL) was respectively performed in triplicate at room temperature. The extracting solvent was then decanted and concentrated by using an R110 Rotavapor (Buchi Labortechnik AG, Flawil, Switzerland) at 40 °C and stored at 4 °C until use (except the water solvent that was removed by freeze drying) (Figure 11).

### 4.2. Cell Lines and Cell Culture

The human colon cancer cell lines (HT-29 and HCT-116), breast adenocarcinoma (MCF-7), lung adenocarcinoma (H23), liver hepatocellular carcinoma (HepG2), cervical cancer (Ca Ski), and normal fibroblast (BJ-5ta) were purchased from the American Type Culture Collection (ATCC, Manassas, VA, USA). Roswell Park Memorial Institute medium (RPMI-1640) supplemented with 10% fetal bovine serum and 1% penicillin and streptomycin (Sigma-Aldrich, St. Louis, MO, USA) was used for cultivation of HCT-116, HT-29, MCF-7, H23, Ca Ski, and BJ-5ta whereas, Dulbecco’s Modified Eagle Medium (DMEM) was used for cultivation of HepG2. All cell lines were cultured in a humidified incubator with 5% CO_2_ at 37 °C.

### 4.3. MTT Cell Proliferation Assay

The MTT assay was carried out based on the previously described method [25]. Briefly, the different cancer cell lines mentioned above (5 × 10^4^ cells/mL) were seeded 24 h prior to treatment in a 96-well plate. Four isolated extracts (hexane, ethyl acetate, methanol, and water) were dissolved in culture medium with different concentration (from 12.5 to 400 μg/mL). Untreated culture medium containing DMSO (0.1%) (Sigma-Aldrich) was served as control negative for entire MTT assay. After incubation of the plates for 24, 48, and 72 h at 37 °C with 5% CO_2_, 20 µL of MTT solution (5 mg/mL; Sigma-Aldrich) was added to each well and then plates were incubated for further 4 hours. A volume of 150 µL DMSO was added to each well and incubated for10 min to dissolve the purple formazan crystals formed at the bottom of the wells. Absorbance was subsequently read at 570 and 650 nm using microplate reader (Asys UVM340, Eugendorf, Austria). Dose-response curves were plotted to obtain IC_50_ values. Each experiment was performed three times [26].

### 4.4. Trypan Blue Assay

To determine cell viability with the trypan blue method 50 μL of 0.4% trypan blue solution was added to 350 μL of media. Then 100 μL of cell stock was added. Four isolated extracts (hexane, ethyl acetate, methanol, and water) were dissolved in culture medium with different concentration (from 12.5 to 400 μg/mL). Untreated culture medium containing DMSO (0.1%) was served as negative control.

### 4.5. Flow Cytometry Analysis

After seeding the MCF-7 cells (5 × 10^5^ cells/well) in a 35 mm dish for 24 h, PVLH-EAE were added to the cells in different final concentration for 48 h. We performed the following sequential actions: (1) detaching the cells with trypsin and EDTA; (2) centrifugation at 7500× *g* for 5 min; (3) washing the pellet cells with PBS containing calcium; (4) DNA staining was carried out and the propidium iodide-stained nuclear fractions were obtained according to the kit protocol. Fluorescence intensity was determined using a FAC Scan flow cytometer (BD Biosciences, San Jose, CA, USA) and analysed by the Cell Quest software.

### 4.6. Acridine Orange/Propidium Iodide Staining (AO/PI)

After 24 h incubation of 1 × 10^6^ cells/well of MCF-7 cells in 6-well plates at 37 °C in a humidified CO_2_ incubator, treatment and control groups were treated with 25 μg/mL PVLH-EAE and 0.1% DMSO for 48 h. Then, the cells were separated with trypsin and AO/PI dyes were added to suspension cells at an equal ratio (10 μL) and were examined under a fluorescence microscope. All viable and early apoptotic cells only uptake the AO dye which bind to double-strand DNA and emit green fluorescence, while PI stained the necrotic and dead cells and look red.

### 4.7. Hoechst 33,342 Staining

Determination and quantification of cell death was based on the classical morphological criteria and together using functional vital dye. Hoechst 33342 is a specific stain for AT-rich regions of double-stranded DNA. This method relies on the differences in the permeability of cell membranes of live, dead and apoptotic cells to the DNA dye, Hoechst 33342. The method is time dependent. Cells were fixed in a solution of 1–3% formaldehyde in PBS at room temperature for 20 min, then the fixing solution were aspirated and cold absolute methanol added, and then were left at room temperature for 20 min. After the methanol aspirated the cell were rinsed thoroughly three times with PBS and incubated for 15 min at 37 °C with Hoechst 33342 dye (5 μg/mL in PBS). The cells are visualized using an Olympus BHZ, RFCA microscope (Tokyo, Japan) equipped with fluorescent light source with an excitation wavelength of 330 nm and a barrier filter of 420 nm. A minimum of 200 cells from the same preparation were counted in five different areas. The experiments are repeated three times and the cell morphology classified according to the following criteria: Live cells :normal nuclei, blue/green pale chromatin with organized structure, apoptotic cells:(early apoptotic cells) can be identified by the presence of chromatin condensation within the nucleus and intact nuclear boundaries, bright blue chromatin that is highly condensed, marginated; (late apoptotic cells) exhibit nuclear fragmentation into smaller nuclear bodies within an intact cytoplasmic membrane.

### 4.8. Gene Expression Assay

Bax and Bcl-2 genes expression were analyzed with real time- PCR. For this purpose, RNA was extracted from approximately 3 × 10^6^ MCF-7 cells, that have been treated with PVLH-EAE, for 24 h using the RNA extraction kit according to the manufacturer’s instructions. The mRNA was transcribed in reverse to cDNA with the Advantage RT-PCR kit using the manufacturer’s protocol. CDNA was amplified using a real time instrument and syber green kit. Specific primers used for amplifying cDNA are presented in Table 3.

### 4.9. Chick Chorioallantoic Membrane Assay

Among the standard methods for evaluating the effects of agents on angiogenesis, we selected the chick chorioallantoic membrane (CAM) assay. Briefly, forty fertilized Ross chicken eggs, purchased from the Toos company (Mashhad, Iran), were carefully cleaned with 70% alcohol and randomly divided into four groups (ten eggs in each group); group 1 was control (without any treatment); and three other groups were experimented (groups 2, 3 and 4 treated with concentrations of 12.5, 25 and 50 µg/mL PVLH-EAE, respectively). Then the eggs were inserted in an automatic rotation Incubator at 38–38.5 °C and 55–65% humidity. After 48 h of the incubation (chick embryo development period), a 1 cm^2^ window was made in the shell under laminar flow hood, next the windows were sealed using sterile paraffin and lamellas, and then the eggs were returned to incubator until day 8 and rotated manually twice a day. On the 8th day of incubation, each specified group was treated with proper concentration of PVLH-EAE under aseptic condition and then, the windows were resealed and the incubation continued for further 72 h. At the day 12 of incubation, the eggs shells were softly removed and CAM were carefully separated and examined with and without microscope. A stereomicroscope (Ziess, Munich, Germany) was used to take photos for analyzing the number and length of blood vessels. The photo was analyzed by the Image J program.

## 5. Statistical Analysis

All values are expressed as mean ± S.D. Student’s *t*-test was used for statistical evaluation of data for non-dose response analysis. Probability values * *p* < 0.05 was considered statistically significant. Dose response assays were analysed using an Omnibus test followed by Rodger’s method.

## 6. Conclusions

This study showed that PVLH-EAE can inhibit the growth of cancer cells and induce apoptosis in human breast cancers in time depended manner. Thus, PVLH-EAE might be most suitable for further research to deal with selective antitumor active substances to human cancers especially breast cancer.

## Figures and Tables

**Table 1:** The IC_50_ (µg/mL) of PVLH different extracts on several cancer cell lines by the MTT assay after 72 h.

Cancer Cell Line	Classification	Hexane Ex.	Ethyl acetate Ex.	Methanol Ex.	Water Ex.
**BJ-5ta**	Normal fibroblast	˃100	˃100	˃100	˃100
**HepG2**	Liver cancer	˃100	92.24 ± 5.82	˃100	˃100
**CasKi**	Cervical cancer	93.44 ± 2.5	81.17 ± 2.87	˃100	˃100
**H23**	Lung cancer	80.31 ± 1.63	67.63 ± 5.54	89.21 ± 1.30	˃100
**MCF-7**	Breast cancer	44.88 ± 1.47	21.20 ± 1.35	˃100	˃100
**HCT116**	Colon cancer	53.20 ± 2.69	25.15 ± 1.85	84.27 ± 1.29	˃100
**HT-29**	Colon cancer	36.17 ± 1.22	23.00 ± 1.2	˃100	˃100

**Table 2:** AO/PI staining of MCF-7 cells.

Group	Time	% Viable	% Apoptotic	% Necrotic
Control MCF-7 + 0.1% DMSO	0	96 + 0.5	0.7 + 0.3	0.3 + 0.2
	24	93.7 ± 2.1	4.5 ± 1.7	1.8 ± 0.7
	48	92.3 ± 1.4	6.8 ± 3.2	2.9 ± 0.5
	72	87.3 ± 0.4	8.9 ± 2.4	4.1 ± 1.1
Treatment MCF-7 + 25 μg/mL PVLH-EAE	0	98.9 + 1.4	0.4 + 0.3	0.7 + 0.4
	24	68.9 ± 1.2	27.87 ± 6.6*	2.1 ± 0.9
	48	49.8 ± 2.6	43.5 ± 3.3*	6.7 ± 1.2
	72	32.1 ± 3.7	57.7 ± 2.7*	8.9 ± 2.5

**Table 3:** Sequence primer.

BCL-2	5 CATGTGTGTGGAGAGCGTCAAC 3 F 5 CAGATAGGCACCCAGGGTGAT 3 R
BAX	5 TTTGCTTCAGGGTTTCATCCA 3 F 5 CTCCATGTTACTGTCCAGTTCGT 3 R
